# Supplementary material for: Chromosome-level Genomes Reveal the Genetic Basis of Descending Dysploidy and Sex Determination in Morus Plants
Source: Genomics Proteomics Bioinformatics. 2022 Aug 30;20(6):1119–37. doi: 10.1016/j.gpb.2022.08.005 (PMC10225493; doi:10.1016/j.gpb.2022.08.005)
Supplement: Supplementary Table S2 [file mmc2.docx]

**Tables S2 Assessment of assemblies**

| **Sample ID** | | **Female *M. notabilis*** | **Male *M. notabilis*** | ***M. yunnanensis*** | ***M.alba*** | ***M.notabilis* (Contig)** |
| --- | --- | --- | --- | --- | --- | --- |
| DNA_mapping | Tissue | Leaf | Leaf | Leaf | * | * |
|  | Bases (bp) | 301,456,058 | 329,129,568 | 313,466,281 | * | * |
|  | Contigs | 542 | 137 | 398 | * | * |
|  | Total_reads | 148,213,457 | 136,903,120 | 140,814,802 | * | * |
|  | Mapped_reads | 145,103,406 | 134,712,658 | 140,107,082 | * | * |
|  |  | 97.90% | 98.40% | 99.50% | * | * |
|  | Tissue | Leaf | Male flower | Leaf | * | * |
| RNA_mapping | Bases (bp) | 301,456,058 | 329,129,568 | 313,466,281 | * | * |
|  | Contigs | 542 | 137 | 398 | * | * |
|  | Total_reads | 42,348,607 | 53,444,632 | 44,174,444 | * | * |
|  | Mapped_reads | 41,531,132 | 47,520,849 | 43,356,759 | * | * |
|  |  | 98.07% | 88.92% | 98.15% | * | * |
| LAI | Chr | whole_genome | whole_genome | whole_genome | whole_genome | whole_genome |
|  | From | 1 | 1 | 1 | 1 | 1 |
|  | To | 301,456,058 | 329,129,568 | 313,466,281 | 336,470,663 | 320,378,613 |
|  | Intact | 0.0494 | 0.0514 | 0.0486 | 0.0441 | 0.015 |
|  | Total | 0.3087 | 0.3091 | 0.3109 | 0.3404 | 0.2575 |
|  | Raw_LAI | 15.99 | 16.62 | 15.62 | 12.95 | 5.81 |
|  | LAI | 20.48 | 19.98 | 21.25 | 18.58 | 8.09 |

*Note*: Asterisk (*) indicates data were not shown in the original article. Chr, chromosome.
