# Supplementary material for: Chromosome-level Genomes Reveal the Genetic Basis of Descending Dysploidy and Sex Determination in Morus Plants
Source: Genomics Proteomics Bioinformatics. 2022 Aug 30;20(6):1119–37. doi: 10.1016/j.gpb.2022.08.005 (PMC10225493; doi:10.1016/j.gpb.2022.08.005)
Supplement: Supplementary Table S3 [file mmc3.docx]

**Tables S3 Repetitive sequences**

| **Sample** |  | **Female *M. notabilis*** | **Male *M. notabilis*** | ***M. yunnanensis*** | ***M. alba*** |
| --- | --- | --- | --- | --- | --- |
| Genome size | Total length | 301,544,460 | 329,129,568 | 313,175,542 | 346,393,484 |
| Repetitive sequence | Total length | 164,410,597 | 192,806,284 | 166,442,299 | 191,454,789 |
| TE sequence | Total length | 148,814,771 | 177,198,293 | 159,993,812 | 175,772,725 |
| SD sequence | Total length | 30,284,480 | 37,379,399 | 31,364,030 | 51,427,933 |
| Retroelements | SINEs | 41,355 | 22,612 | 0 | 10,663 |
|  | LINEs | 1,425,695 | 2,079,263 | 1,615,062 | 2,013,158 |
|  | LTR/*Copia* | 19,665,048 | 23,698,920 | 25,229,701 | 36,723,160 |
|  | LTR/*Gypsy* | 30,126,243 | 28,541,050 | 29,585,987 | 32,905,269 |
| DNA transposons |  | 10,224,769 | 13,080,931 | 10,425,381 | 9,121,416 |
| Satellites |  | 26,203 | 0 | 30,420 | 0 |
| Simple repeats |  | 48,727 | 1,209,713 | 798,089 | 181,741 |

*Note*: TEs, transposable elements; SD, segmental duplication; SINEs, short interspersed elements; LINEs, long interspersed elements.
