# Supplementary material for: Chromosome-level Genomes Reveal the Genetic Basis of Descending Dysploidy and Sex Determination in Morus Plants
Source: Genomics Proteomics Bioinformatics. 2022 Aug 30;20(6):1119–37. doi: 10.1016/j.gpb.2022.08.005 (PMC10225493; doi:10.1016/j.gpb.2022.08.005)
Supplement: Supplementary Table S4 [file mmc4.docx]

**Table S4 Structural variations**

|  |  | ***M. notabilis* *vs*. *M. yunnanensis*** | |  | ***M. notabilis* *vs*. *M. alba*** | |
| --- | --- | --- | --- | --- | --- | --- |
|  |  | **Count** | **Total (bp)** |  | **Count** | **Total (bp)** |
| Insertion | 50–500 bp | 2722 | 353,816 |  | 10,490 | 1,850,421 |
|  | 500–10,000 bp | 945 | 1,312,593 |  | 1767 | 3,491,782 |
|  | 10,000–50,000 bp | 44 | 665,449 |  | 87 | 1,193,921 |
|  | 50,000–100,000 bp | 0 | 0 |  | 0 | 0 |
| Deletion | 50–500 bp | 2394 | 290,382 |  | 6970 | 1,108,501 |
|  | 500–10,000 bp | 458 | 901,217 |  | 1419 | 3,014,470 |
|  | 10,000–50,000 bp | 57 | 1,071,849 |  | 123 | 2,252,486 |
|  | 50,000–100,000 bp | 2 | 139,109 |  | 6 | 437,006 |
| Tandem_expansion | 50–500 bp | 125 | 30,016 |  | 331 | 78,228 |
|  | 500–10,000 bp | 128 | 477,672 |  | 512 | 2,098,217 |
|  | 10,000–50,000 bp | 91 | 2,322,588 |  | 533 | 12,522,323 |
|  | 50,000–100,000 bp | 33 | 1,934,547 |  | 119 | 8,354,696 |
| Tandem_contraction | 50–500 bp | 74 | 17,143 |  | 0 | 0 |
|  | 500–10,000 bp | 52 | 190,136 |  | 0 | 0 |
|  | 10,000–50,000 bp | 11 | 180,805 |  | 0 | 0 |
|  | 50,000–100,000 bp | 0 | 0 |  | 0 | 0 |
| Repeat_expansion | 50–500 bp | 644 | 164,786 |  | 9034 | 1,917,134 |
|  | 500–10,000 bp | 1171 | 3,724,873 |  | 5168 | 11,431,298 |
|  | 10,000–50,000 bp | 236 | 3,950,793 |  | 342 | 5,205,918 |
|  | 50,000–100,000 bp | 4 | 286,731 |  | 1 | 6610 |
| Repeat_contraction | 50–500 bp | 703 | 168,895 |  | 8530 | 1,817,390 |
|  | 500–10,000 bp | 1162 | 3,672,666 |  | 8260 | 22,773,151 |
|  | 10,000–50,000 bp | 418 | 7,825,313 |  | 1906 | 36,632,544 |
|  | 50,000–100,000 bp | 30 | 2,226,463 |  | 141 | 9,707,843 |
| Total number of structural variants | | 11,504 | |  | 55,739 | |
| Total bases affected by structural variants | | 31.91 Mbp | |  | 125.95 Mbp | |
| Total number of SNPs | | 13,179,790 | |  | 89,491,888 | |

*Note*: SNPs, single nucleotide polymorphisms.
