# Supplementary material for: Chromosome-level Genomes Reveal the Genetic Basis of Descending Dysploidy and Sex Determination in Morus Plants
Source: Genomics Proteomics Bioinformatics. 2022 Aug 30;20(6):1119–37. doi: 10.1016/j.gpb.2022.08.005 (PMC10225493; doi:10.1016/j.gpb.2022.08.005)
Supplement: Supplementary Table S6 [file mmc6.docx]

**Table S6 Characteristics of repeated sequences in chromosomal rearrangement regions**

|  | **Size** | **Percent** |
| --- | --- | --- |
| Total length | 1,771,291 bp |  |
| Bases masked | 1,247,440 bp | 70.43% |
| SINEs | 0 bp | 0.00% |
| LINEs | 286 bp | 0.02% |
| LTR elements | 890,769 bp | 50.29% |
| DNA elements | 168,285 bp | 9.50% |
| Unclassified | 133,645 bp | 7.55% |
| Simple repeats | 50,728 bp | 2.86% |
| Low complexity | 10,626 bp | 0.60% |
