# Supplementary material for: Chromosome-level Genomes Reveal the Genetic Basis of Descending Dysploidy and Sex Determination in Morus Plants
Source: Genomics Proteomics Bioinformatics. 2022 Aug 30;20(6):1119–37. doi: 10.1016/j.gpb.2022.08.005 (PMC10225493; doi:10.1016/j.gpb.2022.08.005)
Supplement: Supplementary Table S13 [file mmc13.docx]

**Table S13 CV errors for ADMIXTURE ancestry models with *k* values ranging from 2 to 10**

| **K value** | **CV error** |
| --- | --- |
| K=2 | 0.45414 |
| K=3 | 0.41577 |
| K=4 | 0.40997 |
| K=5 | 0.39637 |
| K=6 | 0.39425 |
| K=7 | 0.37976 |
| K=8 | 0.3766 |
| K=9 | 0.37694 |
| K=10 | 0.37142 |

*Note*: CV, cross-validation.
