# Supplementary material for: Chromosome-level Genomes Reveal the Genetic Basis of Descending Dysploidy and Sex Determination in Morus Plants
Source: Genomics Proteomics Bioinformatics. 2022 Aug 30;20(6):1119–37. doi: 10.1016/j.gpb.2022.08.005 (PMC10225493; doi:10.1016/j.gpb.2022.08.005)
Supplement: Supplementary Table S16 [file mmc16.docx]

**Table S16 Gene amplification and quantitative specific primer sequences**

|  | **Gene** | **Left primer (5'–3')** | **Right primer (5'–3')** |
| --- | --- | --- | --- |
| Gene quantitative | *RECQL1* | ACTTGCCGAATCCATGGAGG | CGATGAGATCTTCGCCGTCA |
|  | *MSTG1* | GCGGACAATTCCGCATCACA | GCTTGGGCGGACTAATTGCT |
|  | *MSTG2* | ATGAACACGATCGGAGGTGA | CCTTCCGACTACTCCTCGTC |
|  | *MSTG3* | CGCTACCGAGATTGCTGACC | GATGTGCATCAGTGGCTGCT |
| Gene amplification | *MSDH*/*RECQL1* | ATGTTATTCCTCCATCATGTTCATGT | TCAATAGTGTTTGTCTTCTACACTGA |
|  | *MSDH*-sp1 | ATGTTATTCCTCCATCATGTTCA | TCAGGTGGAACTCTTGAGACAGTTA |
|  | *MSDH*-sp2 | TCAATAGTGTTTGTCTTCTACACT | CAGCCATTTCTATGCCCAAGA |
|  | *MSTG1* | ATGCGGACAATTCCGCATCA | TCAAAGCGTCAGTCTCAACCTCGAAA |
|  | *MSTG2* | ATGGATCATGTTCCTTCAAA | TCATGACAAAAATAACTGATGAACACG |
|  | *MSTG3* | ATGCTGCGTTGCGTGGTTTCA | CTAGAACAGGTCTTGCTCAATGAAAGT |
