# Supplementary material for: Chromosome-level Genomes Reveal the Genetic Basis of Descending Dysploidy and Sex Determination in Morus Plants
Source: Genomics Proteomics Bioinformatics. 2022 Aug 30;20(6):1119–37. doi: 10.1016/j.gpb.2022.08.005 (PMC10225493; doi:10.1016/j.gpb.2022.08.005)
Supplement: Supplementary Table S17 [file mmc17.docx]

**Table S17 Tissue expression profiles of candidate sex determination genes**

|  | ***MSTG1*** | ***MSTG2*** | ***MSTG3*** | ***RECQL1*** |
| --- | --- | --- | --- | --- |
| Male-root | 0.137673 | 0.9930925 | 0.006319 | 0.3253355 |
|  | 0.137842 | 1.591073 | 0.005514 | 0.3842188 |
|  | 0.116542 | 1.310393 | 0.006715 | 0.3977682 |
| Male-stem | 0.06039 | 0.2222107 | 0.005139 | 0.1855654 |
|  | 0.063849 | 0.6241653 | 0.006264 | 0.1058432 |
|  | 0.092844 | 0.3438855 | 0.00588 | 0.09944206 |
| Male-flower | 0.180076 | 2.265768 | 0.012342 | 0.07536299 |
|  | 0.161693 | 2.056228 | 0.015408 | 0.1182572 |
|  | 0.161592 | 2.297397 | 0.021316 | 0.1267449 |
| Female-stem | 0.000015 | 0.1684042 | 0.000017 | 1.802501 |
|  | 0.00001 | 0.1330463 | 0.000016 | 1.986185 |
|  | 0.000024 | 0.1560413 | 0.000016 | 1.729074 |
| Female-root | 0.001174 | 0.1406323 | 0.000597 | 0.476319 |
|  | 0.000587 | 0.1406323 | 0.000593 | 0.3056601 |
|  | 0.000443 | 0.2448551 | 0.0017403 | 0.196146 |
| Female-flower | 0.000063 | 0.1425955 | 0.00006 | 2.86791 |
|  | 0.000173 | 0.1267449 | 0.000081 | 3.182146 |
|  | 0.000016 | 0.09875517 | 0.000175 | 2.80889 |
